# Long Term Follow up of Patients With Primary Obstetric Antiphospholipid Syndrome

**DOI:** 10.3389/fphar.2022.824775

**Published:** 2022-04-21

**Authors:** Stanley Niznik, Micha J. Rapoport, Orly Avnery, Aharon Lubetsky, Ronen Shavit, Martin H. Ellis, Nancy Agmon-Levin

**Affiliations:** ^1^ Clinical Immunology, Angioedema and Allergy Unit, The Zabludowicz Center for Autoimmune Diseases, Sheba Medical Center, Tel Hashomer, Israel; ^2^ Department of Internal Medicine “C”, Shamir Medical Center, Zerifin, Israel; ^3^ Sackler School of Medicine, Tel Aviv University, Tel Aviv, Israel; ^4^ Hematology Institute and Blood Bank, Meir Medical Center, Kfar Saba, Israel; ^5^ The National Hemophilia Center and Thrombosis Unit, Amalia Biron Research Institute of Thrombosis and Hemostasis, Sheba Medical Center, Tel Hashomer, Israel

**Keywords:** anitphospholipid antibodies, thrombosis, APS - antiphospholipid antibody syndrome, recu recurrence, obstetric APS

## Abstract

**Introduction:** Primary obstetric antiphospholipid syndrome (OAPS) is defined by specific morbidities and/or losses of pregnancy in the presence of persistent antiphospholipid antibodies (aPL). This variant of APS is usually treated during pregnancy and the post-partum period. Data on occurrence of thrombotic event during long term follow-up of OAPS patients is limited.

**Methods:** A multi-centre retrospectively cohort of female patients with primary APS (pAPS) was assembled during 2004–2019. Patients were grouped according to disease presentation as pure OAPS or thrombotic APS (tAPS) for those presenting with thrombosis. Clinical and serological data were compared between groups.

**Results:** Of 219 pAPS female patients 67 (30.6%) were diagnosed with OAPS and 152 (69.4%) with tAPS. During >10 years of follow-up 24/67 (35.8%) OAPS and 71/152 (50%) tAPS suffered a new thrombotic event (*p* = 0.06**)**, while obstetric morbidity was more likely in the OAPS group (31.3 vs. 10.5%, *p* < 0.001) respectively. Among patients with OAPS at presentation heart valve disease and the presence of ANA were related to thrombosis following diagnosis (25 vs. 4.7%, *p* = 0.02; and 45.8 vs. 20.8%, *p* = 0.04 respectively).

**Conclusion:** Thrombotic event following diagnosis were common among female patients with pAPS regardless of disease presentation. Heart valve disease and ANA positivity may be risk factors for thrombosis during follow-up of patients presenting with pure OAPS.

## Introduction

Antiphospholipid syndrome (APS) is defined by obstetric morbidities (i.e., pregnancy losses, intrauterine growth retardation, pre-eclampsia and other partum complications) and/or thrombosis in the persistence presence of antiphospholipid antibodies (aPL’s) (Miyakis et al., 2006). In its primary form (pAPS), APS is not associated with another defined autoimmune disease (e.g., systemic lupus erythematous) although autoantibodies and/or systemic autoimmune manifestations may be present (Gleason et al., 1993; Erkan et al., 2000; Rapoport et al., 2015; Radin et al., 2018). APS in female patients is mostly diagnosed during child bearing age, and a subtype of this syndrome namely obstetric APS (OAPS) is characterized by obstetric morbidity only at presentation (Vinatier et al., 2001; de Jesus et al., 2014; Andreoli et al., 2017). For years treatment of OAPS was recommended only during pregnancy and the post-partum period. Recently, in the European League against Rheumatism (EULAR) guidelines for treatment of APS, low dose aspirin (LDA) and/or prophylactic dose of low molecular weight heparin (LMWH) and/or hydroxychloroquine (HCQ) were recommended for OAPS. Whereas for OAPS patients with high risk aPL profile long term therapy with LDA was suggested to be considered (EULAR, 2020).

The vast majority of studies on OAPS focused on prevention of obstetric complications thereby enabling patients to carry their pregnancies to term without compromising mothers health, (de Jesus et al., 2014; Mekinian et al., 2015; Andreoli et al., 2017; Tektonidou et al., 2019). Data on factors related to the risk of thrombosis in this subgroup of patients is scarce. Moreover, the classification criteria of APS although frequently used for diagnosis of this condition, are less useful to identify risks of further thrombosis. Thus a Global APS score (GAPSS) was developed for this purpose, and incorporates cardiovascular risk factors and aPLs (Sciascia et al., 2013). The adjusted GAPSS (aGAPSS), a simplified version of GAPSS, in which the less available anti- phosphatidylserine/prothrombin (aPS/PT) measurement was excluded, was validated in recent years for assessment of thrombotic recurrence in the general population of APS (Radin et al., 2017; Radin et al., 2019). Nevertheless, assessment of thrombotic risks among patients with pure OAPS is yet an unmet need (Tektonidou et al., 2019). In this study we aimed to provide data on the prevalence and risk factors of thrombotic event during a long term follow-up of patients with OAPS.

## Methods

This is a retrospective study of primary APS patients diagnosed according to the international (Sidney) classification criteria for the antiphospholipid syndrome. Data were retrieved from medical records of sequential patients treated in three large centers in Israel (Sheba-Tel Hashomer, Meir and Shamir Medical Centers) from January 2004 to December 2019. This study was performed in accordance with the declaration of Helsinki and approval of the Institutional Review Boards.

Patients who at presentation of APS or at any point of the disease fulfilled criteria of systemic lupus erythematous disease or another autoimmune/rheumatic disease, based on then relevant classification criteria were excluded. All patients were treated in specialized centers and decisions upon follow up as well as therapeutic ones were at their treating specialist discretion. Given the focus of Obstetric form of APS, only female patients were included in this study.

Demographic characteristics (age, age at diagnosis, length of follow up, treatments); presenting APS classification clinical criteria (i.e., thrombotic or obstetric events and aPL serology); concomitant conditions (hypertension, smoking, diabetes mellitus and dyslipidemia); non-criteria APS-related manifestations manifesting at any time during the disease course (heart valve disease (Libman Sacks endocarditis), livedo reticularis, leg ulcers, migraine, epilepsy, autoimmune hemolytic anemia, thrombocytopenia, leukopenia); APS related outcomes (APS related recurrent events, death, catastrophic APS, aGAPSS, bleeding events) were collected and analyzed.

Patients were divided to two groups.

1) Pure Obstetric APS (OAPS) group constructed of patients with obstetric morbidities only at presentation, alongside consistently elevated antiphospholipid antibody titers (see serology for definition). Obstetric manifestations were defined by: 1) A history of more than 2 recurrent spontaneous miscarriages at week prior to the 10th week of gestation. 2) A history of a spontaneous miscarriage of morphologically normal fetus beyond the 10th week of gestation. 3) A history of one or more premature births of morphologically normal neonate before the 34th week of gestation because of eclampsia/severe preeclampsia or placental insufficiency.

This groups was further divided into two subgroups.

a) Obstetric APS with thrombosis (OAPSt)—patient who were diagnosed with thrombosis after initial diagnosis of OAPS.
b) Obstetric APS without thrombosis (OAPSnt)—patient with no evidence of thrombosis during the follow up period.

2) The thrombotic APS (tAPS) –included female patients with vascular thrombosis at presentation, alongside consistently elevated antiphospholipid antibody titers (see serology for definition)

All patients were followed in their respective clinics, treated by an assigned APS physician. Medical therapy regarded in this study was documented at the time of data collection as along the years multiple changes in treatments were documented. Common doses of treatments were as followed: LDA 75–100 mg/day, LMWH 40–60 mg/day as preventive therapy and 1.5–2 mg/kg/day as a therapeutic dose, Hydroxychloroquine 5 mg\kg per day and prednisone to 5–10 mg prednisone daily.

### Serology and Scores

The presence of anti-cardiolipin (aCL) and anti- β_2_‐glycoprotein I (aβ_2_GPI) of the IgG and IgM isotypes were measured by enzyme‐linked immunosorbent assay (ELISA) or by a multiplex system. Results were considered positive if medium‐to‐high titers (>40 GPL or >20 MPL units [IgG phospholipid units or IgM phospholipid units], which would constitute as the 99th percentile) or according to the manufacturer’s instructions were present in a minimum of two tests performed at least 12 weeks apart were obtained, (Miyakis et al., 2006). Lupus anti-coagulant (LA) activity was detected by coagulation assays in routine use at each center, and was consistent with the International Society of Thrombosis and Hemostasis guidelines. Brandt et al. (1995) aPL positivity was defined as single, double or triple positive according to the number of different positive tests obtained.

In this study we used the aGAPSS (Fernandez Mosteirin et al., 2017; Radin et al., 2017) which allots 3 points for dyslipidemia, 1 point for arterial hypertension, 5 for anti-cardiolipin antibodies IgG/IgM, 4 for anti-β_2_ glycoprotein IgG/IgM and 4 for lupus anticoagulant. Catastrophic APS (cAPS) was defined according to the international task force on CAPS criteria (Cervera et al., 2011).

### Statistical Analysis

The data were analyzed using BMDP software (BMDP Statistical Software, University of California Press, Los Angeles, United States). Pearson’s chi-square test or Fisher’s exact test (two-tailed) was used for analysis of between-group differences in discrete variables, and analysis of variance (ANOVA) was used for comparing continuous variables.

## Results

**FIGURE 1:**
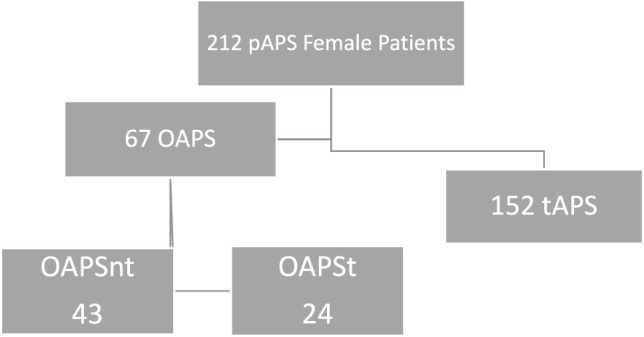
OAPS—Obstetric Antiphospholipid Syndrome, tAPS—Thrombotic Obstetric Syndrome, OAPSnt—Obstetric Antiphospholipid Syndrome with no thrombosis during follow up, OAPSt—Obstetric Antiphospholipid Syndrome with thrombosis during follow up.

In this study 219 APS female APS patients were included, 67 (30.6%) were diagnosed with OAPS, while the remaining 152 (69.4%) presented with a thrombotic event and constructed the group of tAPS (Figure 1).

When comparing patient with OAPS to tAPS (Table 1) during >10 years of follow up the rates of thrombotic occurrence was high in both groups, and did not differ between groups considering arterial or venous events. The composite thrombotic recurrence parameter that included any type of thrombotic recurrence (arterial, or venous or both) was numerically lower in the OAPS group (35.8 vs. 50%; *p* = 0.06). Notably OAPS and tAPS groups did not differ regarding cardiovascular risk factors, aGAPSS (Table 1) or serological parameters (Table 2). In contrast, occurrence of obstetric morbidities during follow up were significantly higher in the OAPS compare to the tAPS group (31.3 vs. 10.5%, *p* < 0.001). Finally, as per assigned therapy, patients with OAPS were more often treated with antiplatelet agent (79.1 vs. 50%, *p* < 0.001) and less likely with therapeutic anticoagulation (32.8 vs. 77%, *p* < 0.001). In a sub group analysis when comparing tOAPS with pregnancy, at any point in their life time up to the time of data collection (*n* = 118), with tOAPS with no pregnancy (*n* = 34), no major differences were seen in regards of age of presentation, cardiovascular risk factors, aGAPSS score, type of thrombosis, rates of thrombotic recurrence or type of therapy.

**TABLE 1:** Clinical parameters of OAPS vs. tAPS female patients.

Parameter	OAPS (*n* = 67)	tAPS (*n* = 152)	*p* Value
Age	43 (±13.8)	46.8 (±10.3)	
General Patients parameters and cardiovascular risk factors			
Age at presentation (years)	33.2 (±13.7)	36.2 (±7.7)	0.09
Follow up time (years)	9.8 (±7.6)	10.6 (7.7)	0.5
Hypetension	7 (10.5%)	28 (18.4%)	0.14
Smoking	8 (11.5%)	15 (9.8%)	0.65
Dyslipidemia	7 (10.5%)	19 (12.5%)	0.7
aGAPSS	10.6 (±3.6)	10.9 (±3.6)	0.6
Non Manifestation Criteria			
Heart Valve Disease	8 (11.9%)	18 (11.8%)	0.99
Livedo Reticularis	6 (8.6%)	13 (9%)	0.9
Auto Immune Haemolytic Anaemia	10 (14.9%)	19 (12.5%)	0.7
Thrombocytopenia	16 (23.9%)	7 (21.7%)	0.7
Recurrence			
Any form of recurrence	41 (61.19%)	76 (50%)	0.14
Obstetric	21 (31.3%)	16 (10.5%)	<0.001
Arterial	15 (22.4%)	41 (27%)	0.5
Venous	5 (7.46%)	19 (12.5%)	0.3
Combined	4 (6%)	11 (7.2%)	0.7
Composite Thrombotic Recurrence	24 (35.8%)	71 (50%)	0.06
Therapy			
Antiplatelet	53 (79.1%)	76 (50%)	<0.001
Anticoagulation	22 (32.8%)	117 (77%)	<0.001
Combination of antiplatelet and therapeutic Anticoagulation	11 (16.4%)	39 (25.6%)	0.6
Hydroxychloroquine	17 (25.4%)	42 (27.6%)	0.7
Glucocorticoids	1 (1.5%)	15 (9.9%)	0.03
General Outcome			
CAPS	1 (1.5%)	4 (2.6%)	0.6
Mortatlity	0 (0%)	5 (3.3%)	0.14
Bleeding	5 (7.5%)	16 (10.5%)	0.5

**TABLE 2:** Serological parameters of OAPS vs. tAPS female patients.

Parameter	OAPS (*n* = 67)	tAPS (*n* = 152)	*P* value
Anti-B2GPI (IgM)	34 (50.8%)	79 (52%)	0.4
Anti-B2GPI (IgG)	39 (58.2%)	96 (63.1%)	0.9
Anti-cardiolipin (IgM)	31 (46.3%)	72 (47.4%)	0.6
Anti-cardiolipin (IgG)	46 (68.7%)	102 (67.1%)	0.9
Lupus anti-coagulant	42 (62.7%)	108 (71.1%)	0.15
aPL Triple positive	34 (50.8%)	83 (54.6%)	0.7
ANA positive	20 (29.85%)	50 (32.89%)	0.6
C3 (mean value) mg/dl	111 (±33)	114 (±30)	0.7
C4 (mean value) mg/dl	24 (±11)	22.7 (±11.2)	0.5

Focusing on the OAPS group of which approximately a third of patients suffered from thrombotic events (OAPSt) during follow up. The latter included arterial 54.2% (13/24) venous 25% (6/24) and combined events (i.e., both arterial and venous) 20.9% (5/24). While comparing OAPSt group to OAPS patients with no-thrombosis during follow up the OAPSnt group, few differences were noted (Table 3). Mainly heart valve disease was diagnosed in 25% (6/24) of OAPSt vs. 4.7% (2/43) of OAPSnt patients (*p* = 0.02). Overall cardiovascular risk factors did not differ between groups although hypertension and dyslipidaemia were numerically higher (20.8 vs. 4.7% *p* = 0.09 for both parameters). Serological comparison between OAPSt and OAPSnt groups (Table 4) suggested an association between the presence of antinuclear antibody and OAPSt (45.8 vs. 20.8%, *p* = 0.04 respectively). Interestingly, neither aPLs, nor triple positivity differ between groups.

**TABLE 3:** Clinical parameters of OAPS patients with thrombosis (OAPSt) and without thrombosis during follow up (OAPSnt).

Parameter	OAPSt (*n* = 24)	OAPSnt (*n* = 43)	*p* value
Age (years)	47.5 (±11.8)	40.7 (±7.4)	0.001
Demographics and cardiovascular risk factors			
Age at presentation (years)	35.5 (±8.8)	31.8 (±6.7)	0.05
Follow up Time (years)	11.9 (±7.6)	9.4 (±6.7)	0.03
Hypertension	5 (20.8%)	2 (4.7%)	0.09
Smoking	3 (11.6%)	5 (12.5%)	0.9
Diabetes	1 (2.3%)	1 (4.2%)	0.7
Dyslipidemia	5 (20.8%)	2 (4.7%)	0.09
aGASPSS	11.6 (±3.2)	10 (±3.5)	0.07
Non criteria Manifestation			
Heart Valve Disease	6 (25%)	2 (4.7%)	0.02
Livedo Reticularis	1 (4.1%)	5 (11.6%)	0.4
Autoimmune Hemolytic Anemia	3 (12.5%)	1 (2.3%)	0.13
Thrombocytopenia	3 (12.5%)	13 (30.2%)	0.14
Bleeding	3 (12.5%)	2 (4.7%)	0.34
Therapy			
Antiplatelet	16 (66.7%)	37 (86%)	0.12
Anticoagulation	18 (75%)	4 (9.3%)	<0.001
Combination of Antiplatelet and therapeutic anticoagulation	12 (50%)	3 (7%)	<0.001
Hydroxychloroquine	7 (29.2%)	10 (23.2%)	0.8
Glucocorticoid	1 (4.2%)	0 (0%)	0.36

**TABLE 4:** Serological Clinical parameters of OAPS patients with thrombosis (OAPSt) and without thrombosis during follow up (OAPSnt).

Parameter	OAPSt (*n* = 24)	OAPSnt (*n* = 43)	*p* Value
Anti-B2GPI (IgM)	54.1% (13)	48.8% (21)	0.8
Anti-B2GPI (IgG)	54.1% (13)	60.5% (26)	0.8
Anti-cardiolipin (IgM)	54.1% (13)	41.9% (18)	0.33
Anti-cardiolipin (IgG)	54.1% (13)	72.1% (31)	0.2
Lupus anti-coagulant	66.6% (16)	58.1% (25)	0.5
aPL Triple positive	58.3% (14)	46.5% (20)	0.35
ANA positive	45.8% (11)	20.9% (9)	0.04
C3 (mean value) mg/dl	20.8% (5)	11.6% (4)	0.2
C4 (mean value) mg/dl	16.6% (4)	9.3% (2)	0.09

## Discussion

In this real life retrospective analysis of 212 female patients, 30.6% patients were diagnosed with pure OAPS at presentation, of whom 35.8% subsequently suffered a thrombotic event.

Thrombotic occurrence in OAPS patients, is of great interest as well of practical importance. Previously published studies suggest that pure OAPS might hint to different mechanisms in OAPS in comparison to thrombotic-APS (Meroni et al., 2012; Meroni et al., 2018; Bettiol et al., 2020). In this line of thought a significant difference was observed in our cohort in regard to rates of recurrent obstetric events which were significantly higher in OAPS compare to thrombotic-APS (31.3 vs. 10.5%, *p* < 0.001).

In regard to thrombosis following the diagnosis of OAPS the evidence is discordant between studies, as some suggest that the rate of thrombosis is low (Alijotas-Reig et al., 2015; Jiang et al., 2021), while others documented a high risk despite preventive therapy (Lefèvre et al., 2011; Drozdinsky et al., 2017). One such example is derived from The APS ACTION cohort in which the risk of thrombosis amounted to 60% among OAPS patient (de Jesús et al., 2019). In our study the rate of thrombosis was within this spectrum as 35.8% of OAPS patients experienced a least one thrombotic event during 10 years of follow up. Moreover rates of different type of thrombosis (arterial or venous) were similar between patients with OPAS compare to female patients with thrombotic-APS at presentation. Differences in the prevalence of thrombosis in OPAS and particularly in the current study may result from amalgamation of two parameters on the one hand the relative high rate of triple positivity while on the other is the low rate of classical cardiovascular risk factors in our cohort. Another plausible explanation is differences between populations as our cohort derived only of Israeli patients. Intriguingly, one may suggest that our data hint to OAPS thrombotic risks also in the absence of cardiovascular risk factors. Although differences were observed between cohorts it seems that the risk of thrombosis in OAPS remain substantial. Of note, current studies and guidelines, strongly recommend the use of antiplatelet therapy as primary prophylaxis in pregnant patients with positive APL antibodies (Andreoli et al., 2017; Tektonidou et al., 2019). We believe this study, stresses the importance of such recommendation in light of risk of thrombosis in OAPS.

Additionally, two risk factors for thrombosis in OAPS patients were observed in our cohort namely the presence of heart valve disease and ANA sero-positivity. This stands in agreement with the APS ACTION cohort, in which thrombosis was associated with heart valve disease (de Jesús et al., 2019). In the same line also in an Argentinian study non-criteria manifestation were linked with thrombosis in OAPS (Udry et al., 2019). Studies have addressed the discrepancies between criteria classification (which are used for clinical studies) (Öztürk et al., 2004), while non criteria manifestation, as in this study, are of growing importance in real life setting. As for ANA positivity, such an association was previously reported in a combined cohort of primary and secondary APS patient (Lefèvre et al., 2011). Our report is the first to the best of our knowledge the first to describe this association in primary APS patient. Interestingly, none of our patients that demonstrated ANA positivity was diagnosed with SLE or other defined autoimmune disease within the study period, according to the ACR criteria as well as the new EULAR/ACR classification criteria (EULAR, 2020).

The aGAPSS is a useful tool to predict thrombosis in the general population of APS patients (Ruiz-Irastorza et al., 2011; Chighizola et al., 2018; Garcia and Erkan, 2018), alike is the presence of multiple aPL (i.e., triple positivity) (Pengo et al., 2010; Udry et al., 2019). Notably in our study both parameters were numerically higher 11.6 vs. 10 for aGAPSS and 58.3 vs. 46.5% for triple aPL positivity in OAPSt vs. OAPSnt respectively. While both differences did not reach statistical significance a trend can be suggested. This lack of statistical differences may be attributed to several factors such as the size of our cohort, the relatively high triple positivity of aPL in our entire cohort (>50%) as well as the plausible lower rate of cardiovascular risk factors assessment in a cohort of female patients. On the other hand in this cohort, among OAPS patients with no cardiovascular risk factors, the rate of thrombosis was similarly high, alluding to a plausible role of other factors not included in the aGAPSS. Taking it all together is seems that emphasizing the need to assess and treat hypertension, dyslipidaemia and other cardio-vascular risk factors in APS patient in general and OAPS in particular is of the essence.

Our study withhold some limitations derived from its retrospective nature as incomplete and possible bias reporting especially given the prolonged duration of follow up. The lack of documentation of adherence to therapy, and the multiple treatment changes along more than 10 years of follow up. Moreover, defining timing of the thrombosis and re-thrombosis was equivocal, thus it was difficult to establish how many of the thrombotic event were during per partum, and which therapy was given. Lastly, a relatively high rate of thrombosis was documented in our cohort compare to previously published cohort (Cervera et al., 2015; de Jesús et al., 2019), particularly in patients with tAPS. This difference may derive from the nature of our relatively high risk cohort (>50% of patients presented with triple aPL positivity), the length of follow up and/or geographic variation (Israeli patient population). However, we believe that these limitations are compensated for by the multicenter nature of this study which is relatively large and the well-defined cohort of primary APS female patients.

## Conclusion

In this retrospective study, we have showed that risk for thrombosis following APS diagnosis in females is high regardless of presenting symptom. As more than a third of female patients with OAPS suffered a least one arterial or venous thrombotic event during follow up, that did not differ in comparison to female patients that presented with thrombotic-APS (tAPS group). In our OAPS cohort the main risk factors for subsequent thrombosis were heart valve disease and the presence of ANA positivity, while cardiovascular risk factors, aGAPSS and triple aPLs positivity were numerically higher in this sub-group of patients. High index of suspicion for these risk factors may enable physicians to improve their management and decrease the rate of new thrombotic events in OAPS.

## Data Availability

The original contributions presented in the study are included in the article/Supplementary Material, further inquiries can be directed to the corresponding author.
